# Deciphering the deterministic role of TCR signaling in T cell fate determination

**DOI:** 10.3389/fimmu.2025.1562248

**Published:** 2025-05-21

**Authors:** Zhen Qin, Tao Xu

**Affiliations:** Zhongshan School of Medicine, Sun Yat-sen University, Guangzhou, China

**Keywords:** TCR signaling, T cell fate, T cell differentiation, TCR signal strength, immunometabolism, T cell exhaustion, Tpex, memory T cells

## Abstract

T cell receptor (TCR) signaling, also known as signal 1, plays a crucial role in the activation and proliferation of T cells. The question of whether TCR signaling exerts a deterministic role in T cell fate determination is an area of active investigation. It has been particularly challenging to address this question due to the complexities associated with genetic manipulation of TCR signaling components, which often disrupts thymic T cell development or impairs T cell activation upon TCR engagement. Recent study demonstrates that the TCR-Lck/Fyn axis directly induces STAT3 phosphorylation and synergizes with pro-inflammatory cytokines to optimize STAT3 phosphorylation during Th17 cell differentiation. Additionally, the TCR-Lck/Fyn-AKT/mTOR axis negatively regulates Treg cell differentiation. In CD8^+^ T cells, persistent high-affinity antigen stimulation drives differentiation along the exhaustion pathway, while acute infection or intermediate antigen levels promote differentiation into effector and memory T cells, although the underlying mechanism remains to be fully elucidated. Collectively, these studies provide compelling evidence that TCR signaling has a deterministic impact on T cell fate. This review summarizes recent advances in understanding how TCR signaling shapes T cell fate determination.

## Introduction

The activation and differentiation of T cells need 3 signals: signal 1 provided by the interaction between T cell receptor (TCR) and peptide-MHC complex presented by APCs; signal 2 mediated by the interaction between CD28 expressed on T cells and CD80/86 on APC cells; and signal 3 provided by cytokines in the microenvironment (1). TCR specifically recognizes short peptide antigens (8–17 amino acids) presented by major histocompatibility complex (MHC) molecules on the surface of antigen-presenting cells (APCs) (2–4). TCR engagement with pMHC triggers a cascade of intracellular signaling leading to the activation of transcription factors such as NFATs, Jun/AP-1, and NF-κBs which translocate into the nucleus to drive the expression of many genes, such as IL-2, IL-2rα, generally essential for the activation and proliferation of all the T cells (5). The signal 2, is also called “co-stimulation signal” provided primarily through CD28 binding to CD80 or CD86 on APCs, are indispensable for enhancing T cell survival and proliferation. Other co-stimulatory receptors, such as ICOS, 4-1BB, and OX40, also contribute to fine-tuning the T cell activation (6, 7). Conversely, co-inhibitory receptors like CTLA-4 and PD-1 counterbalance these signals, regulating the magnitude of T cell activation and preventing immune overactivation (8). The signal 3, mediated by cytokines, directs the differentiation of activated T cells into different lineages (9). For CD4^+^ T cells, cytokines such as IL-12 and IL-4 promote Th1 and Th2 differentiation via the transcription factors T-bet and GATA-3, respectively, while TGF-β and inflammatory cytokines like IL-6 and IL-23 drive Th17 polarization through RORγt (10). In CD8^+^ T cells, cytokines like IL-12 and type I interferons enhance effector differentiation, while IL-15 is essential for memory formation (11). The coordinated interplay among these three signals ensures that T cells differentiate into specialized effector and memory populations tailored to the specific antigenic challenge, providing both immediate immune defense and long-term protection.

However, recent studies suggest that TCR signaling may not merely serve as an on/off switch for T cell activation; rather, it appears to provide nuanced signals that influence the specific lineage commitment of T cells (12). For instance, the strength and duration of TCR signaling can dictate whether CD4^+^ T cells differentiate into Th1, Th2, Th17, or regulatory T cell (Treg) subsets, each of which plays a distinct role in the immune response (13). Attenuation of TCR signaling favors the differentiation of activated CD4^+^ T cells into Treg cells (14–16). Similarly, CD8^+^ T cell differentiation into effector or memory subsets is also modulated by TCR signaling strength and duration (17). All these studies suggest that TCR signaling may play a deterministic role in T cell fate determination. Here in this review, we will summarize the recent evidence that TCR signaling itself plays a key deterministic role in T cell fate determination, as well as the underlying mechanism. Deep understanding of how TCR signaling influence T cell fate determination is crucial, particularly in the development of therapeutic strategies aimed at manipulating T cell responses in diseases such as cancer and autoimmune diseases. By deciphering the complex interplay between TCR signals, co-stimulatory signals, and the cytokine environment, researchers can potentially identify novel targets for immunotherapy. Such strategies could enhance T cell responses against tumors, improve vaccine efficacy, or even restore tolerance in autoimmune diseases.

## Overview of TCR signaling

TCR is a heterodimer composed of TCRα and TCRβ chains, each containing variable and constant regions (18). TCR is associated with the CD3 subunits consisting of invariant γ, δ, ϵ, and ζ subunits responsible for transducing intracellular signals following antigen recognition, which together are called as TCR-CD3 complex (19). Upon recognizing specific peptide antigens presented by major histocompatibility complex (MHC) molecules on antigen-presenting cells (APCs), the TCR undergoes conformational changes that lead to exposure of the immunoreceptor tyrosine-based activation motifs (ITAMs, YXXM motifs) located in the cytoplasmic tails of the CD3 subunits (20), facilitating phosphorylation of the tyrosines within these motifs by Src-family protein tyrosine kinases, predominantly Lck and Fyn (21, 22). Phosphorylated ITAM motifs recruit ZAP-70 kinases, which facilitates its phosphorylation by Lck/Fyn kinases (23). Phosphorylated ZAP-70 then phosphorylates key adaptor proteins, including Linker for Activation of T Cells (LAT) and SH2 domain-containing leukocyte protein of 76 kDa (SLP-76), which form signaling complexes (24). LAT and SLP-76 provide docking sites for multiple effector proteins, enabling the activation of downstream pathways such as the MAPK/ERK pathway, the phosphatidylinositol 3-kinase (PI3K)/AKT/mTOR pathway, and the NF-κB pathway, which trigger a series of intracellular signaling cascades that lead to T cell activation, and proliferation (25, 26).

These pathways play critical roles in metabolic reprogramming and cell survival. For instance, the mTOR pathway integrates signals from TCR engagement, costimulatory receptors, and nutrient availability to regulate T cell fate decisions, including effector and memory differentiation (27). While mTOR is often activated via the canonical PI3K-AKT pathway, it can also be triggered independently of AKT through mechanisms such as amino acid sensing mediated by Rag GTPases (28, 29). PI3K activation leads to the phosphorylation of AKT, which further amplifies mTOR activity, supporting T cell growth, proliferation, and metabolic adaptation (30, 31). Meanwhile, LAT and SLP-76 also facilitate the recruitment of phospholipase C-γ1 (PLC-γ1), which catalyzes the hydrolysis of phosphatidylinositol 4,5-bisphosphate (PIP2) into diacylglycerol (DAG) and inositol 1,4,5-triphosphate (IP3) (32). DAG activates protein kinase Cθ (PKCθ) and RasGRP1, which promote the activation of the NF-κB and MAPK pathways, respectively (33). IP3, on the other hand, induces the release of intracellular calcium from the endoplasmic reticulum, leading to the activation of the phosphatase calcineurin and the subsequent nuclear translocation of the transcription factor NFAT (nuclear factor of activated T cells) (34).

These signaling pathways collectively activate key transcription factors, including NFAT, AP-1 (activator protein 1), and NF-κB. These transcription factors together orchestrate the transcriptional programs necessary for T cell activation, proliferation, differentiation, and effector function (35, 36). The role of LAT in assembling these signaling complexes is critical, as mutations in LAT disrupt T cell development and cytokine production, thereby impairing immune responses (37–41).

While the role of TCR signaling in T cell activation is well established, increasing evidence supports its substantial influence on T cell fate decisions. Rather than being merely permissive, TCR signaling contributes instructively to lineage commitment by modulating transcriptional and metabolic programs. Although many studies have demonstrated causal links between TCR signal strength and specific differentiation outcomes—particularly in CD4^+^ and CD8^+^ subsets—important questions remain regarding how these signals are integrated across different cellular and environmental contexts. Continued investigation is necessary to fully decipher the precise mechanisms and to determine how TCR signaling interacts with co-stimulatory, cytokine, and metabolic cues to guide long-term fate decisions. Deepening our understanding of these pathways holds significant implications for the development of targeted immunotherapies in cancer, chronic infection, and autoimmune diseases (33).

## TCR signaling and CD4^+^ T cell fate determination

CD4^+^ T cells are pivotal components of the adaptive immune system, capable of differentiating into specialized functional subsets—such as Th1, Th2, Th17, T follicular helper (Tfh), and Tregs—depending on the signals they encounter. These lineages are defined by transcription factors and unique cytokine profiles, and adopt distinct roles. Th1 cells drive cellular immunity against intracellular pathogens, Th2 cells promote humoral immunity to combat extracellular parasites, Th17 cells defend against extracellular bacteria and fungi, and Tregs maintain immune tolerance (42). The differentiation of CD4^+^ T cell lineages is highly context-dependent, requiring the integration of three critical signals: TCR signaling (signal 1), co-stimulatory signals (signal 2), and cytokine signals (signal 3). The strength, duration, and quality of TCR signaling are crucial factors influencing lineage commitment and functional specialization.

## TCR signal strength and lineage commitment

The strength and duration of TCR signaling are critical determinants in T cell fate decisions, influencing the differentiation of naive CD4^+^ T cells into distinct effector or regulatory subsets (13, 43, 44). Recent studies find that the intensity of TCR signaling plays a pivotal role in directing lineage commitment, supporting a deterministic model where stronger signals drive effector T cell differentiation while weaker signals promote the development of regulatory T cells (45).

Strong TCR signaling typically favors the differentiation of Th1, Th17 and Tfh cells through the activation of lineage-defining transcription factors (10, 45–50). For instance, robust and sustained TCR signals promote Th1 differentiation by enhancing the expression of T-bet, the master transcription factor for Th1 cells (51), which drives the production of IFN-γ, a key cytokine involved in cellular immunity against intracellular pathogens (52). Likewise, the TCR-Lck/Fyn axis facilitates STAT3 activation, supporting Th17 differentiation (12). Similarly, strong TCR signals, in conjunction with costimulatory molecules and IL-21 signaling, promote the expression of Bcl-6, the transcription factor essential for Tfh cell differentiation, which supports the generation of high-affinity antibodies in germinal centers (53–55).

Expanding on the role of TCR signal quality, a recent study dissected the distinct contributions of antigen affinity and antigen dose in shaping CD4^+^ T cell differentiation during infection (56). The results demonstrated that high-affinity peptide-MHC interactions preferentially promote Th1 differentiation, independent of antigen dose, whereas Tfh cells can arise across a broader range of affinities but require sustained antigen availability to persist. Notably, increasing the antigen dose could not compensate for the suboptimal Th1 differentiation induced by low-affinity peptides. Furthermore, memory CD4^+^ T cells retained recall potential shaped by the strength of the initial TCR signal, emphasizing how early TCR engagement imprints long-term functional bias. These findings highlight antigen affinity as a critical determinant in effector subset specification and memory imprinting, with important implications for vaccine design and T cell-based immunotherapies.

Strong TCR signals have been shown to inhibit default Th2 differentiation programs by preventing early IL-4 expression and autocrine signaling through GATA3, thereby promoting Th1 over Th2 differentiation (57, 58). Studies suggest that this process is mediated by the nuclear translocation of NFATp and alterations in the DNA binding activity of AP-1 under strong TCR signaling (59, 60). This mechanism prevents IL-4-mediated feedback loops that would otherwise promote Th2 polarization, effectively guiding the cell toward a Th1 phenotype under conditions of robust antigen engagement.

**Figure 1:**
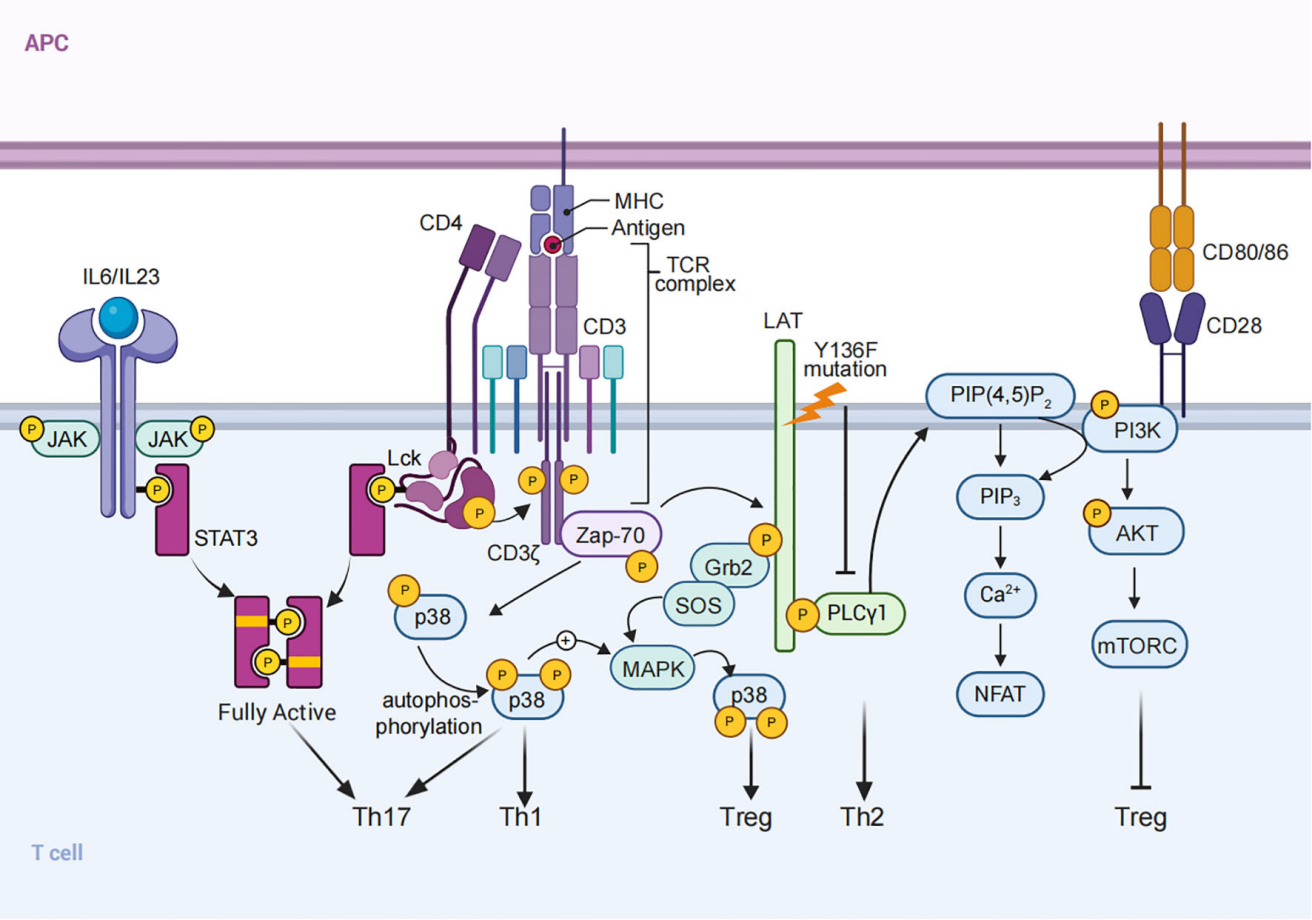
TCR Signaling and CD4^+^ T Cell Differentiation. Schematic representation of TCR signaling in CD4^+^ T cell activation and differentiation. Signal 1 (TCR signaling), Signal 2 (co-stimulation), and Signal 3 (cytokine signals) integrate to regulate T cell fate. Examples include: (1) the TCR-Lck/Fyn axis synergizing with cytokines to optimize STAT3 phosphorylation, promoting Th17 differentiation; (2) LAT mutations that alter COOH-terminal tyrosine residues lead to defective signaling, resulting in Th2-skewed cytokine responses; and (3) PI3K/AKT/mTOR signaling, activated by TCR stimulation, inhibits FOXP3 expression and suppresses Treg differentiation. (4) p38 MAPK signaling, activated via ZAP70 and LAT-SOS, promotes Th1 and Th17 differentiation, while also modulating Treg development.

A key component in this regulatory process is the adaptor protein LAT (Linker for Activation of T cells), which functions as a scaffold, facilitating the assembly of the “LAT signalosome,” a multiprotein complex that organizes and links TCR signals to intracellular pathways such as MAPK and NF-κB, thereby influencing lineage commitment (41). While LAT has been primarily understood as a positive regulator of TCR signaling, recent findings reveal its dual role. Studies of LAT mutations in mouse models, where the COOH-terminal tyrosine residues of LAT are altered, have shown that defective LAT signaling can lead to lymphoproliferative disorders characterized by polyclonal T cells with increased Th2 cytokine production (61). This unexpected finding underscores LAT’s role as a negative regulator of excessive TCR signaling, helping maintain T cell homeostasis and limiting unwarranted Th2 differentiation (**Figure 1**).

In addition, recent studies have identified two distinct pathways for p38 MAPK activation in T cells (1): a classical MAPK cascade mediated by LAT-SOS, and (2) an alternative pathway involving direct phosphorylation by ZAP70 (62–64). Basal ZAP70 activation primes the classical p38 pathway, lowering the activation threshold and facilitating full p38 activation upon TCR engagement. This dual activation mechanism plays a critical role in maintaining immune balance, as evidenced by impaired IL-2 production and reduced Treg differentiation in the absence of Sos1 and Sos2. Furthermore, p38 MAPK signaling influences CD4^+^ T cell differentiation, with its inhibition leading to decreased Th1, Th17, and inducible Treg (iTreg) differentiation (**Figure 1**). These findings underscore the complex interplay between TCR signaling strength, adaptor proteins, and downstream kinase pathways in shaping T cell fate (64, 65).

TCR signal strength has differing implications for thymus-derived (tTreg) and peripherally induced (pTreg) subsets (14, 66, 67). Tregs, essential for maintaining immune tolerance and preventing autoimmune responses, are characterized by the expression of the transcription factor FOXP3 (68–70). tTregs are typically generated in response to moderate-to-high affinity TCR interactions with self-antigens during T cell development in the thymus (71–73). This engagement drives FOXP3 expression and establishes a stable epigenetic program, reinforced by histone modifications such as H3K4me2 and H3K4me3 at the Foxp3 loci, which prevents lineage deviation into effector T cells (66, 74–77). In contrast, the generation of pTregs in peripheral tissues involves relatively weak TCR signaling, often in response to environmental antigens or commensal microbes, rather than high-affinity self-antigens (14). Weaker TCR signals, especially when combined with anti-inflammatory cytokines like TGF-β and IL-2, promote FOXP3 induction in peripheral T cells (78, 79). This allows pTregs to modulate immune responses in peripheral tissues, providing a flexible mechanism for maintaining tolerance to non-self antigens encountered outside the thymus​ (15, 66, 80, 81).

Distinct intracellular pathways underscore these differences. In pTregs, weak TCR stimulation is thought to favor FOXP3 induction partly by reducing PI3K/AKT/mTOR signaling, which would otherwise antagonize FOXP3 expression (15, 82). For instance, reduced activity of the PIP3 phosphatase PTEN—a regulator of AKT signaling—is associated with suboptimal TCR stimulation, promoting FOXP3 expression in pTregs (80, 83) (**Figure 1**). Additionally, adaptor molecules like ITK, which are activated downstream of TCR engagement, modulate PTEN activity; ITK deficiency enhances pTreg differentiation, indicating that reduced signaling through this pathway may support FOXP3 stability in pTregs (80). In tTregs, continuous TCR signaling is generally unnecessary for maintaining FOXP3 expression once cells have fully differentiated, as evidenced by studies where TCRα deletion in mature Tregs does not lead to loss of identity of Treg cells in a resting state, although its deletion may cause functional impairment (84, 85).

Recent findings on TCR signaling complexity provide additional insights into how signal strength and duration affect Treg differentiation. The TCR-CD3 complex contains a total of 10 ITAMs, with each CD3ζ chain contributing three ITAMs (six in total from the CD3ζ homodimer), and the remaining four derived from the CD3γ, CD3δ, and two CD3ϵ subunits (20). ITAM multiplicity amplifies TCR signaling, critical for certain specialized T cell functions requiring strong or sustained TCR–ligand interactions. However, studies using knock-in mice expressing non-signaling CD3ζ chains (6Y within ITAM motifs were mutated into 6F) suggest that ITAM multiplicity is not essential for general T-cell functions like cytokine production or the development of a diverse antigen-reactive TCR repertoire (48). However, the knock-in mice exhibited greatly increased cell number of Treg cells. These findings imply that while strong ITAM-mediated signals may be vital for tTreg differentiation, weaker ITAM signaling could suffice for pTreg induction, particularly in the presence of supportive cytokine environments.

Furthermore, pharmacological interventions such as the use of rapamycin analogs (e.g., everolimus) and Srci1 (highly selective inhibitor of Lck/Fyn) enhance pTreg generation by inhibiting AKT/mTOR pathways and stabilizing FOXP3 expression through reduced DNA methylation at the Foxp3 promoter and CpG island (86, 87). Nutrient-sensing pathways, such as Rag GTPase-dependent mTORC1 activation, have also been implicated in the functional programming of Tregs, especially activated Tregs in peripheral tissues and tumors (88). These pathways link metabolic cues to Treg expansion and suppressive capacity, further emphasizing the interplay between TCR signaling, cytokines, and metabolism in Treg biology.

Under Th17-polarizing conditions, strong and sustained TCR signaling, characterized by high antigen dose and persistent stimulation, has been shown to promote IL-17 expression, the hallmark cytokine of Th17 cells (12, 89, 90), while moderate TCR stimulation, typically involving lower-affinity antigens or reduced antigen availability, tends to favor Treg development over Th17 polarization, even in the presence of similar cytokine environment (14, 80). However, the precise role of TCR signal strength in determining the balance between Th17 and Treg differentiation remains complex and the underlying mechanism is still to be determined. Our recent studies demonstrates that the TCR-Lck/Fyn axis directly phosphorylates STAT3 at Y705, synergistically with proinflammatory cytokines like IL-6 and IL-23, to achieve the optimal STAT3 phosphorylation needed for Th17 lineage commitment. Pharmacological inhibition of Lck/Fyn kinase activity, or disrupting its interaction with STAT3, significantly reduces STAT3 phosphorylation, skewing differentiation away from the Th17 pathway and toward a Treg phenotype (91). Mechanistically, Lck and Fyn interact with STAT3 as evidenced by results from co-immunoprecipitation assays. AlphaFold Multimer analysis indicates that the MAS-motif within STAT3 initiates the interaction of between STAT3 and Lck/Fyn, enhanced their kinase activity, and is essential for its phosphorylation by Lck/Fyn, which is further supported by *in vitro* kinase assay showing that the peptide containing WT MAS motif significantly enhanced STAT3 phosphorylation by Lck kinases. This critical interaction demonstrates the deterministic role of TCR signaling in directing Th17 differentiation. Notably, disruption of the interaction between Lck/Fyn ad STAT3 by disease causing STAT3 mutation selectively inhibits TCR stimulation induced STAT3 phosphorylation, but not proinflammatory cytokines induced STAT3 phosphorylation, and inhibits Th17 cell differentiation, which further demonstrates the significance of TCR-Lck/Fyn-STAT3 axis in Th17 cell differentiation. Administration of the Src inhibitor Srci1 or disruption of the Lck/Fyn-STAT3 interaction significantly ameliorated experimental autoimmune encephalomyelitis (EAE), a Th17-mediated autoimmune disease, by reducing Th17 differentiation and enhancing Treg polarization. These findings not only demonstrate modulation of the TCR-Lck/Fyn-STAT3 axis holds promise for therapeutic intervention, but also uncover the critical synergy between TCR signaling and cytokine networks (Signal 3) in regulating CD4^+^ T cell differentiation, with the TCR-Lck/Fyn axis serving as a key determinant of Th17 lineage fate (**Figure 1**).

## Integration of TCR signaling with cytokine networks

The integration of TCR signaling with cytokine networks is critical for directing CD4^+^ T cell lineage commitment and plasticity. CD4^+^T cells differentiation is orchestrated by a dynamic interplay between intrinsic TCR signals and extrinsic cytokine inputs, which collectively shape T cell fate and function. Strong TCR signals combined with cytokines like IL-12 and IFN-γ promote Th1 differentiation by activating STAT1 and inducing T-bet expression, whereas IL-4 signaling favors Th2 lineage commitment through STAT6 and GATA3 activation (87, 92–95). In the context of Th17 differentiation, IL-6 and TGF-β act in concert with TCR signaling to activate STAT3, driving the differentiation process, which engage STAT3 and RORγt, the master transcription factor for Th17 cells, thereby reinforcing lineage-specific gene expression (12, 90, 96–99). Notably, TCR-Lck/Fyn axis directly induces STAT3 phosphorylation, establishing a critical link between TCR strength and cytokine-driven lineage determination during Th17 cell differentiation (12).

The plasticity of CD4^+^ T cells enables functional adaptation in response to changing cytokine environments, disruptions in the cytokine-TCR signaling axis can lead to immune dysregulation. For example, Th1 cells reactivated in Th2-polarizing conditions can express Th2 cytokines, demonstrating the dynamic nature of T cell responses (100, 101). Similarly, Tregs and Th17 cells exhibit reciprocal plasticity influenced by the balance of TGF-β and pro-inflammatory cytokines such as IL-6 and IL-23 (102). This plasticity highlights the importance of a finely tuned cytokine milieu in shaping T cell fate even after initial differentiation. These cytokines and TCR signaling pathways converge to influence CD4^+^ T cell fate, underscoring the coordination required between extracellular signals and intracellular transcriptional regulation.

In summary, TCR signaling plays a critical and multifaceted role in the differentiation of CD4^+^ T cells into distinct functional subsets. The context in which TCR signaling occurs—particularly the cytokine environment and the strength of the signal—dictates whether CD4^+^ T cells differentiate into Th1, Th2, Th17, or Treg subsets. Recent advances in understanding the molecular mechanisms underlying TCR signaling, such as the role of the TCR-Lck/Fyn-STAT3 axis and the MALT1 cleavage pathway (12, 103), have provided deeper insights into how TCR signaling influences immune function and tolerance. Understanding these mechanisms is crucial for developing targeted immunotherapies aimed at modulating T cell responses in autoimmune diseases, infections, and cancer.

## Integration of TCR signaling with metabolic networks

Metabolic reprogramming occurs upon T cell activation, with metabolism and nutrient cues reciprocally influencing TCR signaling strength and T cell differentiation. Upon TCR engagement, T cells shift from oxidative phosphorylation (OXPHOS) to aerobic glycolysis, a phenomenon known as the Warburg effect (108, 109). This metabolic switch facilitates the rapid generation of ATP and metabolic intermediates essential for proliferation and effector functions (110). The mechanistic mTOR pathway, a central regulator of metabolism, links TCR signaling to metabolic reprogramming, controlling glycolysis, lipid synthesis, and amino acid metabolism to promote effector T cell differentiation (88, 104). Intrinsic cellular energy demands are synchronized with extracellular environmental signals, such as nutrient availability and pH, to ensure proper T cell activation, proliferation, and differentiation (105, 106). Given the growing recognition of metabolism’s pivotal role in T cell differentiation, it is now often referred to as “signal 4.”

Distinct CD4^+^ T cell subsets exhibit unique metabolic dependencies. Effector T cells, such as Th1 and Th17 cells, rely on glycolysis, and amino acid metabolism (105), whereas Tregs favor oxidative phosphorylation and fatty acid oxidation (FAO) to support their suppressive function (106–108). This metabolic divergence is crucial for lineage specification, reinforcing how TCR signaling and metabolic programs coordinate to shape T cell fate.

Beyond glycolysis and amino acid metabolism, sterol metabolism has emerged as a key regulator of TCR signaling and CD4^+^ T cell differentiation (109). Upon TCR engagement, activated T cells upregulate sterol biosynthesis and uptake pathways to support membrane expansion during clonal proliferation (110). The liver-X receptors (LXRs), which serve as metabolic sensors, regulate this process (111, 112). Notably, LXRβ deficiency enhances both CD4^+^ and CD8^+^ T cell proliferation, leading to increased IFN-γ production, highlighting the metabolic influence on effector function (111).

Metabolically, sterol metabolism directly influences lineage decisions. Activation of LXRs suppresses Th17 differentiation via sterol regulatory element-binding protein-1 (SREBP-1), which competes with the aryl hydrocarbon receptor (AhR) for binding at the Il17a locus, thereby repressing Th17 lineage commitment (113, 114). In addition, a cholesterol biosynthetic intermediate has been demonstrated as endogenous Rorγt ligand to direct Th17 differentiation (115). These findings suggest that sterol metabolism not only supports proliferation but also modulates differentiation by regulating lineage-defining transcriptional programs.

## TCR signaling in CD8^+^ T cell fate determination

TCR signaling plays a pivotal role in orchestrating the differentiation and functional specialization of CD8^+^ T cells, influencing their development, differentiation, and functional responses, also determining the long-term behavior of CD8^+^ T cells. CD8^+^ T cells are essential for immune defense against viral infections and tumors, and their function is closely linked to the nature and strength of TCR signals received during antigen recognition. A growing body of evidence has illuminated how TCR signaling intricately modulates CD8^+^ T cell differentiation, particularly the balance between short-lived effector cells and long-lived memory cells, as well as the risk of T cell exhaustion (116, 117).

## TCR Signal Strength and Duration Shape CD8⁺ T Cell Fate

High-affinity TCR interactions are essential for the differentiation of cytotoxic effector T cells (CTLs), which directly target infected or malignant cells (118). Strong, sustained TCR signals—often amplified by co-stimulatory molecules like CD28—activate transcription factors such as T-bet and Eomesodermin (Eomes) (119, 120). These factors orchestrate the expression of cytotoxic machinery, including perforin and granzymes, which is crucial for immediate pathogen elimination. Studies indicate that high-affinity TCR engagement is a primary driver of robust CTL differentiation, where prolonged TCR engagement has been shown to enhance terminal effector differentiation, equipping CD8^+^ T cells with rapid and potent immune capabilities.

In contrast to the differentiation of effector cells, intermediate TCR signals tend to favor the formation of memory CD8^+^ T cells, which provide long-term protection by rapidly responding to subsequent antigen exposure (116, 121, 122). Memory T cells exhibit distinct metabolic and functional profiles, enabling their prolonged survival and capacity for robust recall responses (123). This balance between persistence and responsiveness ensures effective immunity against reinfections (124). In addition to promoting effector functions, strong TCR signals have also been shown to modulate the mode of cell division in activated CD8^+^ T cells (125). Under strong stimulation, asymmetric cell division (ACD) safeguards memory potential by enabling fate bifurcation within progeny, whereas symmetric divisions favor terminal effector differentiation. Inhibiting ACD under high TCR signaling impairs memory generation, suggesting that ACD acts as a regulatory mechanism that preserves long-term immunity under conditions of intense stimulation.

Moreover, the duration of TCR signaling has also been shown to directly influence memory T cell formation (123). Shorter TCR signaling durations lead to the generation of memory precursors, which maintain the ability to rapidly proliferate and acquire effector functions upon secondary antigen encounters. This dichotomy in TCR signaling strength provides a finely tuned mechanism by which CD8^+^ T cells balance their immediate effector functions with long-term memory formation, ensuring both rapid pathogen clearance and durable immunity (126).

In addition to governing the balance between effector and memory T cell differentiation, TCR signal strength also plays a decisive role in the development of exhausted CD8^+^ T cells (Tex) and progenitor exhausted T cells (Tpex) (127, 128). Factors such as peptide-MHC affinity, contact time with dendritic cells (DCs), persistent antigen load, and the number of antigen-presenting DCs determine TCR signal strength during priming (129). High TCR signal strength has been shown to increase the expression of inhibitory receptors such as PD-1 and LAG-3, driving terminal exhaustion and reduced cytotoxic function in chronic infections and tumors (130). Conversely, lower TCR signal strength favors the generation of Tpex cells, a less differentiated subset that retains proliferative capacity and responds better to immune checkpoint blockade (ICB) therapy (131–134). This highlights the need to optimize TCR signaling thresholds to balance protective immunity, persistence, and responsiveness to immunotherapy.

## Regulatory mechanisms and external modulation of TCR signaling

One of the critical aspects of TCR signaling is its ability to dictate the expression of transcription factors that govern CD8^+^ T cell differentiation. For instance, the transcription factor interferon regulatory factor 4 (IRF4) is essential for the expansion and differentiation of CD8^+^ T cell. Recent studies demonstrated that the expression of IRF4 in CD8^+^ T cells is contingent upon the strength of TCR signaling, which is partially mediated by mTOR signaling pathways (135). This finding corroborated another study which further elucidated that graded levels of IRF4 regulate CD8^+^ T cell differentiation and expansion in response to acute viral infections (136), highlighting the importance of TCR signaling in this context. Additionally, the role of IL-2 inducible T-cell kinase (ITK) in regulating IRF4 expression underscores the complexity of TCR signaling pathways and their downstream effects on CD8^+^ T cell fate (119).

CD45, a critical phosphatase, plays a significant role in modulating TCR sensitivity in naive and memory CD8^+^ T cells (137). Continuous interaction with self-MHC ligands is crucial for the survival of naive T cells but not for memory cells, indicating distinct TCR sensitivity between these subsets (138). High CD45 expression in memory CD8^+^ T cells is associated with reduced TCR sensitivity compared to naive cells. This reduced sensitivity, linked to decreased activation of LCK and short-term TCR signaling, protects CD8^+^ T cells from excessive auto-MHC reactivity while preserving robust responses to foreign antigens (139). This differential regulation highlights how TCR signaling mechanisms adapt to the distinct functional requirements of naive and memory T cell populations.

The role of cytokines in modulating TCR signaling cannot be overlooked. Pathogen-specific inflammatory environments can enhance TCR signaling in CD8^+^ T cells, thereby tuning their antigen sensitivity and functional responses (140). This interplay between cytokine signaling and TCR engagement is crucial for effective differentiation, as transient enhanced IL-2 receptor signaling during priming amplifies the development of functional effector-memory cells (141). Furthermore, both TCR and IL-2 signaling strength control memory CD8^+^ T cell functional fitness via chromatin remodeling, highlighting the integration of external cues with intrinsic signaling (142).

Beyond antigen recognition, co-stimulation, and cytokine signaling, emerging evidence suggests that metabolic cues act as signal 4 in T cell activation, influencing TCR signaling strength, CD8^+^ T cell differentiation, and functional persistence (143–145). TCR engagement induces metabolic reprogramming, shifting from oxidative phosphorylation to glycolysis to meet the bioenergetic and biosynthetic demands of rapid expansion and effector differentiation (146). CD28 co-stimulation further enhances glycolysis, amplifying metabolic flux to support proliferation and cytokine production (147). Notably, high glycolytic activity favors effector differentiation but impairs the long-term survival of memory CD8^+^ T cells (148, 149). Thus, balancing glycolysis with fatty acid oxidation (FAO) is critical for sustaining effective immune responses (150).

Metabolic constraints in the tumor microenvironment (TME) further modulate TCR signaling strength and CD8^+^ T cell functionality (151). In nutrient-deprived conditions, such as low glucose and amino acid availability, CD8^+^ T cells upregulate alternative metabolic pathways to sustain their function (152). However, chronic metabolic stress can impair TCR signaling, driving metabolic exhaustion and dysfunction (153). Notably, glycolysis is directly linked to TCR-dependent IFN-γ production, as reducing glycolytic activity dampens cytokine output and cytotoxicity (154). Conversely, selectively enhancing glycolysis restores effector functions and may serve as a strategy to reinvigorate exhausted CD8^+^ T cells in immunotherapy settings (155).

Additionally, the mTOR pathway plays a critical role in linking TCR signaling to metabolic adaptation (104). Increased glycolytic activity activates mTOR, promoting effector differentiation but potentially contributing to metabolic dysregulation and partial T cell dysfunction (28). Interestingly, mTOR-dependent metabolic shifts may adversely affect IFN-γ production in some contexts (156), further emphasizing the necessity of fine-tuned metabolic control. Furthermore, metabolic competition between glycolysis and FAO influences the long-term survival and recall capacity of memory CD8^+^ T cells (157, 158). While effector cells rely on glycolysis for rapid cytotoxic responses, memory cells preferentially utilize FAO, allowing for extended persistence and robust recall responses upon reinfection (159).

TCR signaling is further modulated by the acidic conditions of the TME, where extracellular acidification suppresses T cell function (160). Acidosis directly impacts T cell metabolism by restricting one-carbon metabolism, limiting nucleotide biosynthesis and impairing activation potential. Additionally, low pH inhibits TCR signal transduction via the STS1-Cbl-b complex, a pH-sensitive phosphatase that actively suppresses T cell function. This regulatory mechanism constrains CD8^+^ T cell effector responses in tumors, dampening anti-tumor immunity. Notably, the deficiency of either STS1 or Cbl-b desensitizes T cells to acidic pH, leading to enhanced anti-tumor reactivity and improved T cell survival in hostile microenvironments. These findings underscore the metabolic constraints imposed by acidic niches and their impact on TCR-driven fate decisions.

Metabolic adaptations under nutrient-deprived conditions also modulate TCR signaling strength and CD8^+^ T cell differentiation (154). In the TME, where glucose and amino acid availability are limited, CD8^+^ T cells upregulate nutrient transporters and alternative metabolic pathways to sustain functionality (152, 155). However, chronic metabolic stress can impair TCR signaling and drive T cell dysfunction, ultimately promoting T cell exhaustion, a hallmark of dysfunctional tumor-infiltrating lymphocytes (153). Understanding these adaptations provides therapeutic opportunities to enhance anti-tumor immunity by modulating metabolic pathways to optimize TCR function in tumors.

In addition to biochemical and metabolic inputs, mechanical forces have emerged as a novel regulatory dimension of TCR signaling (161–163). T cells apply cytoskeletal tension at the immunological synapse to interrogate antigen-presenting cells, and TCR-pMHC interactions can behave as catch bonds that prolong signal duration under force (162). These mechanosensing mechanisms enhance antigen discrimination and influence downstream transcriptional programs, adding an underexplored but important layer of control to T cell activation and fate decisions.

Taken together, TCR signaling and metabolism are highly interdependent. Targeting metabolic pathways in conjunction with TCR signaling holds promise for enhancing T cell-based immunotherapies, improving T cell persistence, cytotoxicity, and survival in cancer and chronic infections (158, 159).

## Developmental dynamics and long-term outcomes

The dynamics of TCR signaling play a pivotal role in CD8^+^ T cell differentiation (164, 165). These findings underscore the importance of TCR signaling not only in immediate immune responses but also in shaping long-term immune potential.

**Figure 2:**
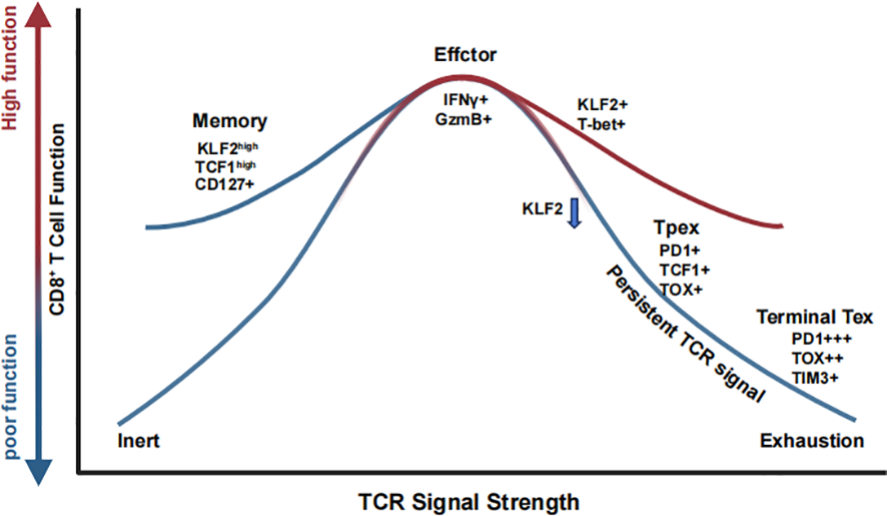
TCR signal strength shapes CD8^+^ T cell fate through coordinated transcriptional and functional programs. CD8^+^ T cells interpret graded TCR signals to adopt distinct functional states. Low TCR stimulation promotes memory precursor formation (KLF2^+^ TCF1^+^ CD127^+^), supporting long-term persistence. Intermediate TCR strength induces optimal effector differentiation (IFN-γ^+^ GzmB^+^) with sustained KLF2 and T-bet expression. Prolonged or excessive TCR signaling drives progressive loss of KLF2 and acquisition of exhaustion-associated markers (PD-1, TOX), leading to a stepwise transition from progenitor exhausted T cells (Tpex) to terminally exhausted T cells (Tex).

A seminal study challenged the traditional paradigm that only strong TCR ligation is sufficient to initiate T cell responses (166). Their findings demonstrated that even very low-affinity TCR–peptide-MHC interactions can activate naïve CD8^+^ T cells, induce rapid proliferation, and generate both effector and memory populations. Notably, while low-affinity interactions were sufficient to trigger early activation and migration—marked by faster CCR7 downregulation and earlier tissue egress—sustained T cell expansion required higher-affinity stimulation. This differential expansion contributes to a maturation of the T cell pool over time, favoring the prolonged persistence of high-affinity clones. Interestingly, despite limited expansion, low-affinity ligands were still capable of generating functional memory T cells, indicating that memory formation is not strictly dependent on strong TCR signals (**Figure 2**). The clonal composition of responding CD8^+^ T cells is shaped by TCR avidity thresholds, which govern recruitment and expansion (167). High-avidity T cells dominate protective responses, but low-avidity clones also contribute functional flexibility, especially during heterologous re-infections. This layered recruitment enhances the adaptability of T cell responses, with clonal diversity tuned by the affinity landscape of peptide-MHC interactions during priming. TCR signal strength influences both the extent of effector responses and the qualitative diversity of memory and clonal selection.

Recent innovative studies have further elucidated the role of TCR signaling in determining CD8^+^ T cell fate. Evidence now suggests that precursor exhausted T (Tpex) cells arise early in both acute and chronic infections, positioning them at a critical bifurcation point where they can either differentiate into memory precursors following pathogen clearance or progress toward terminal exhaustion under conditions of persistent antigen exposure (165). This finding emphasizes that the pMHC-TCR signal itself is a decisive factor in dictating whether CD8^+^ T cells differentiate into memory T cells or exhausted T cells, irrespective of infection type (168). Moreover, a groundbreaking Perturb-seq study identified Klf2 as a key transcription factor governing CD8^+^ T cell fate determination (169) (**Figure 2**). The study demonstrated that Klf2 knockout drives aberrant differentiation toward an exhaustion-like state even in acute infections, suggesting that Klf2 plays an essential role in suppressing the exhaustion-promoting transcription factor TOX while enabling T-bet to drive effector differentiation. Additionally, strong TCR stimulation was found to silence Klf2 at protein level. These findings reinforce the concept that TCR signaling is the primary determinant of CD8^+^ T cell differentiation, directly influencing exhaustion versus effector/memory lineage commitment.

Chronic antigen stimulation during persistent infections or cancer can lead to T cell exhaustion, characterized by diminished effector function and upregulation of inhibitory receptors such as PD-1, Tim-3, and LAG-3 (170–172). Recent studies suggest that TCR signal strength is a key determinant of exhaustion dynamics in CD8^+^ T cells (130, 173) (**Figure 2**). High-affinity, persistent TCR signaling promotes terminal exhaustion, whereas lower TCR signal intensity favors the differentiation of Tpex cells, which retain proliferative potential and responsiveness to immune checkpoint blockade therapy (127, 174, 175). Recent findings further elucidate that the formation of Tpex cells is initiated early during acute responses and is driven by strong TCR signaling and high-affinity peptide-MHC interactions. This developmental trajectory is counterbalanced by PD-1 signaling, which restricts precursor expansion (165). Together, these findings reinforce the idea that TCR signal strength not only governs early activation but imprints long-term lineage potential across both effector and memory trajectories.

Interestingly, both low- and high-affinity antigen-expressing tumors exhibit resistance to immune control, albeit through different mechanisms (176). High-affinity tumors induce deep exhaustion by promoting prolonged antigen stimulation and inhibitory receptor expression, leading to dysfunctional T cells. Conversely, low-affinity tumors fail to elicit strong TCR engagement, resulting in poor CD8^+^ T cell priming and weak immune responses. These findings suggest that fine-tuning TCR signaling—both during early priming in lymphoid tissues and within the tumor microenvironment—could optimize antitumor immunity (**Figure 2**). This principle also applies to adoptive T cell therapies, where selecting optimal TCRs or chimeric antigen receptors (CARs) based on antigen affinity and signal strength may improve T cell persistence, functionality, and therapeutic efficacy (177–180).

In addition to its role in memory formation and exhaustion, TCR signaling strength has also been implicated in tissue-resident memory T cell (TRM) differentiation (181). TRM cells are non-circulating CD8^+^ T cells that provide long-term localized immunity across various tissues (182). Recent evidence suggests that lower TCR signal strength favors TRM differentiation, while high-affinity TCR interactions preferentially drive circulating memory T cell formation (183). In models of influenza virus infection, weaker TCR stimulation correlated with enhanced TRM formation in the lung, whereas stronger TCR signaling biased cells toward a circulating effector memory phenotype (184).

Beyond antigen affinity, DC subsets play a crucial role in shaping TRM differentiation by influencing TCR signaling strength during priming (185). Recent studies have shown that mice lacking DNGR1 or Batf3—key markers of cross-presenting DCs—exhibited impaired TRM formation after viral infection, while circulating memory T cells were unaffected (186). These findings highlight the interplay between TCR strength, antigen-presenting cell specialization, and tissue-specific cues in determining whether CD8^+^ T cells persist in circulation or take up long-term residence within tissues.

Moreover, TCR strength influences the chemotactic properties of CD8^+^ T cells, regulating their ability to establish tissue residency (183). Weaker TCR signaling is associated with reduced expression of KLF2 and S1PR1 (187, 188), which promotes TRM retention by limiting their egress from tissues. These observations suggest that TCR signaling not only governs differentiation into effector versus memory states but also fine-tunes tissue-specific localization and persistence. TCR signal strength also governs chemotactic behavior, including the upregulation of CXCR6 and suppression of S1PR1 expression, promoting tissue residency. This chemotactic switch is dependent on Blimp1 induction by TCR re-stimulation and is essential for efficient TRM differentiation across non-lymphoid tissues (183). These findings emphasize how TCR signal strength not only instructs fate commitment but also spatial positioning of effector progeny during immune responses.

Sustained TCR signaling is also known to drive this exhausted phenotype by inducing the expression of transcription factors such as TOX and NR4A, which suppress effector functions and promote inhibitory receptor expression (189). Exhausted CD8^+^ T cells exhibit reduced cytotoxic capacity, impairing their ability to clear chronic infections or tumors effectively. Importantly, recent therapeutic strategies have focused on reversing T cell exhaustion by blocking inhibitory receptors with immune checkpoint inhibitors, thus restoring the effector functions of exhausted CD8^+^ T cells in the context of cancer immunotherapy. These approaches highlight the critical importance of understanding TCR signaling dynamics in addressing chronic diseases and cancer.

TCR signaling is a fundamental determinant of CD8^+^ T cell fate, shaping their development, differentiation, and functional potential. The strength, duration, and context of TCR signals, coupled with co-stimulatory inputs and metabolic programming, govern the balance between effector differentiation, memory formation, and the risk of exhaustion. These signals influence whether CD8^+^ T cells adopt an effector or memory phenotype or become exhausted under chronic antigen exposure. A deeper understanding of these mechanisms is essential for developing targeted therapies to enhance CD8^+^ T cell responses in infections, cancer, and chronic diseases. Future research into the nuances of TCR signal modulation will be critical for advancing immunotherapeutic strategies, ultimately improving outcomes across diverse clinical applications.

In conclusion, TCR signaling plays a critical role not only in T cell activation but also in fate determination. As we unravel the complex signaling networks involved in T cell differentiation, we move closer to developing therapies that can precisely modulate T cell responses in diverse disease contexts. Continued exploration of TCR signaling nuances will undoubtedly yield innovative therapeutic strategies, transforming the landscape of T cell based immunotherapy.
